# Supplementary material for: Biologically Active Echinulin-Related Indolediketopiperazines from the Marine Sediment-Derived Fungus Aspergillus niveoglaucus
Source: Molecules. 2019 Dec 23;25(1):61. doi: 10.3390/molecules25010061 (PMC6983058; doi:10.3390/molecules25010061)

Biologically active echinulin-related indolediketopiperazines from marine sediment-derived fungus *Aspergillus niveoglaucus*

Olga F. Smetanina [1], Anton N. Yurchenko [1*], Elena V. Girich (Ivanets) [1], Phan Thi Hoai Trinh [2], Alexander S. Antonov [1], Sergey A. Dyshlovoy [1,3,4,5], Gunhild von Amsberg [4,5], Natalya Yu. Kim [1], Ekaterina A. Chingizova [1], Evgeny A. Pislyagin [1], Ekaterina S. Menchinskaya [1], Ekaterina A. Yurchenko [1], Tran Thi Thanh Van [2] and Shamil Sh. Afiyatullov[1]

[1] G.B. Elyakov Pacific Institute of Bioorganic Chemistry, Far Eastern Branch of the Russian Academy of Sciences, Prospect 100-letiya Vladivostoka, 159, Vladivostok 690022, Russia;
smetof@rambler.ru (O.F.S.); ev.ivanets@yandex.ru (E.V.I.); pibocfebras@gmail.com (A.S.A.); dyshlovoy@gmail.com (S.A.D.); natalya_kim@mail.ru (N.Y.K.); pislyagin@hotmail.com (E.A.P.); ekaterinamenchinskaya@gmail.com (E.S.M.); dminae@mail.ru (E.A.Y.); afiyat@piboc.dvo.ru (S.S.A.)

[2] Department of Marine Biotechnology, Nhatrang Institute of Technology Research and Application, Vietnam Academy of Science and Technology, 650000 Nha Trang, Vietnam;
phanhoaitrinh84@gmail.com (P.T.H.T.); tranthanhvan@nitra.vast.vn (T.T.T.V.)

[3] School of Natural Science, Far Eastern Federal University, Sukhanova St., 8, Vladivostok 690000, Russia

[4] Laboratory of Experimental Oncology, Department of Oncology, Hematology and Bone Marrow Transplantation with Section Pneumology, Hubertus Wald-Tumorzentrum, University Medical Center Hamburg-Eppendorf, 20246 Hamburg, Germany;
g.von-amsberg@uke.de (G.A.)

[5] Martini-Klinik Prostate Cancer Center, University Hospital Hamburg-Eppendorf, 20246 Hamburg, Germany

* Correspondence: yurchant@ya.ru; Tel.: +7-423-231-1168

Content

Table S1. Cytotoxic activities of isolated compounds 1-7 3
Urease inhibition activity 3
Table S2. 1D and 2D NMR spectroscopic data of cryptoechinuline B (1). 4
Figure S1. ^{1}H NMR spectrum (700 MHz, DMSO-d_6) of cryptoechinulin B (1) 6
Figure S2. ^{13}C NMR spectrum (176 MHz, DMSO-d_6) of cryptoechinulin B (1) 7
Figure S3. DEPT-135 spectrum (176 MHz, DMSO-d_6) of cryptoechinulin B (1) 8
Figure S4. HSQC spectrum (700 MHz, DMSO-d_6) of cryptoechinulin B (1) 9
Figure S5. HMBC spectrum (700 MHz, DMSO-d_6) of cryptoechinulin B (1) 10
Figure S6. ^{1}H-^{1}H COSY spectrum (700 MHz, DMSO-d_6) of cryptoechinulin B (1) 11
Figure S7. ROESY spectrum (700 MHz, DMSO-d_6) of cryptoechinulin B (1). 12
Figure S8. CD spectrum of (+)-cryptoechinulin B (1a) and (–)-cryptoechinulin B (1b) 13

Table S1. Cytotoxic activities of isolated compounds 1-7

Compounds	Cytotoxicity IC_{50}, μM			
	Neuro-2a	22Rv1	PC-3	LNCaP
**1a**	> 100	-	-	-
**1b**	> 100	-	-	-
**2**	> 100	-	-	-
**3**	> 100	63.2	41.7	25.9
**4**	50.9	-	-	-
**5**	40.6	-	-	-
**6**	> 100	-	-	-
**7**	> 100	49.9	63.8	38.9
**Docetaxel (reference drug)**	n/t	0.013	0.015	0.004

Urease inhibition activity

Compounds **3–7** were tested in a cell-free urease activity assay. In this assay echinulin (**3**) showed activity with an IC_{50} of 29.8 μM, while the other compounds were inactive up to concentration of 100 μM. Thiourea used as positive control inhibited urease activity with an IC_{50} of 23 μM.

Urease (urea amidohydrolase EC 3.5.1.5) is an enzyme responsible for the hydrolysis of urea into ammonia and CO2 or carbamate [1]. Activity of this enzyme has been implicated in the pathogenesis of several human diseases [2]. The most known examples of urease producing pathogenic bacteria are Helicobacter pylori and Mycobacterium tuberculosis [3]. Thus, urease are among of the promising pharmacological targets in the search for new antibiotics [2,4]. Moderate urease inhibition by echinulin (3) observed by us was in line with the previous report by Du et al., which describes an antibacterial activity of echinulin (3) against Staphylococcus aureus [5]. Thus, urease inhibition could be a possible mechanism which stipulates echinulin antimicrobial properties.

Urease Inhibition Assay

The reaction mixture consisting of 25 μl enzyme solution (urease from *Canavalia ensiformis*, Sigma, 1U final concentration) and 5 μl of test compounds dissolved in water (0.2 - 100.0 μM final concentration) was preincubated at 37 °C for 60 min in 96-well plates. Then 55 μl of phosphate buffer solution with 100 mM urea was added to each well and incubated at 37 °C for 10 min. The urease inhibitory activity was estimated by determining of ammonia production using indophenol method [6]. Briefly, 45 μL of phenol reagent (1% w/v phenol and 0.005% w/v sodium nitroprusside) and 70 μL of alkali reagent (0.5% w/v NaOH and 0.1% active chloride NaOCl) were added to each well. The absorbance was measured after 50 min at 630 nm using a microplate reader Multiscan FC (Thermo Scientific, Canada). All the reactions were performed in triplicate in a final volume of 200 μL. The pH was maintained 7.3-7.5 in all assays. DMSO 5% was used as a positive control.

1. Bano, B.; Kanwal; Khan, K.M.; Lodhi, A.; Salar, U.; Begum, F.; Ali, M.; Taha, M.; Perveen, S. Synthesis, in vitro urease inhibitory activity, and molecular docking studies of thiourea and urea derivatives. *Bioorg. Chem.* **2018**, *80*, 129-144.
2. Rutherford, J.C. The Emerging Role of Urease as a General Microbial Virulence Factor. *PLOS Pathogens* **2014**, *10*, e1004062.
3. Mobley, H.L. The role of *Helicobacter pylori* urease in the pathogenesis of gastritis and peptic ulceration. *Aliment. Pharmacol. Therapeut.* **1996**, *10*, 57-64.
4. Hameed, A.; Anwar, A.; Khan, K.; Malik, R.; Shahab, F.; Siddiq, S.; Zahra Basha, F.; Choudhary, M. Urease inhibition and anticancer activity of novel polyfunctional 5,6-dihydropyridine derivatives and their structure-activity relationship. *Eur. J. Chem.* **2013**, *4*, 49-52.
5. Du, F.Y.; Li, X.M.; Li, C.S.; Shang, Z.; Wang, B.G. Cristatumins A-D, new indole alkaloids from the marine-derived endophytic fungus *Eurotium cristatum* EN-220. *Bioorg. Med. Chem. Lett.* **2012**, *22*, 4650-4653.
6. Weatherburn, M.W. Phenol-Hypochlorite Reaction for Determination of Ammonia. *Anal. Chem.* **1967**, *39*, 971-974.

Table S2. 1D and 2D NMR spectroscopic data of cryptoechinuline B (1).

№	*δ*C, type	*δ*H (*J* in Hz)	HMBC
NH (1)		10.87, s	2, 3, 3a, 7a
2	143.8, C		
3	103.3, C		
3a	124.2, C		
4	117.1, CH	7.03, d (7.8)	3a, 6, 7a
5	120.3, CH	6.80, dd (8.2, 1.5)	3a, 7, 20
6	134.0, C		
7	110.6, CH	7.18, brs	3a, 5, 20
7a	135.4, C		
8	111.1, CH	6.94, s	2, 3a, 9, 10
9	124.1, C		
10	161.3, C		
NH (11)		7.67, s	9, 10, 13
12	59.1, C		
13	167.6, C		
NH (14)		8.73, s	10, 12
15	38.9, C		
16	145.1, CH	6.05, dd (17.5, 10.5)	2, 15,18,19
17	111.5, CH_2	5.02, dd (10.9, 1.2)<br>4.99, dd (17.9, 1.2)	15, 16<br>15, 16
18	27.5, CH_3	1.45, s	15, 16, 19
19	27.5, CH_3	1.43, s	15, 16, 18
20	33.8, CH_2	3.36, d (7.5)<br>3.36, d (7.5)	5,6, 7, 21, 22<br>5,6, 7, 21, 22
21	124.1. CH	5.32, brt (7.6)	20, 23, 24
22	130.9, C		
23	17.5, CH_3	1.71, s	21, 22, 24
24	25.4, CH_3	1.71, s	21, 22, 23
25	37.6, CH_2	a: 2.1, dd (13.5, 7.1)<br>b: 1.69, dd (13.5, 7.2)	12, 26, 27, 29<br>12, 13, 26, 29, 44
26	28.3, CH	2.68, m	
27	132.7, CH	5.74, dt (10.2, 2.7)	26, 28, 29
28	124.7, CH	5.63, dt (10.9, 3.4)	12, 26, 27, 29
29	45.5, CH	3.56, m	28
30	137.0, CH	5.85, dd (15.8, 8.3)	27, 28, 29, 31
31	124.4, CH	6.71, d (16.1)	29, 37, 32
32	117.2. C		
33	128.5, C		
34	153.0, C		
35	124.3, C		
36	125.0, CH	6.99, s	34, 35, 37, 38
37	146.9, C		
38	26.7, CH_2	3.21, d (7.5)<br>3.21, d (7.5)	33, 34, 36, 39, 40<br>33, 34, 36, 39, 40
39	121.4, CH	5.23, brt (7.5)	41, 42
40	132.6, C		
41	17.5, CH_3	1.64, s	39, 40, 42
42	25.5, CH_3	1.70, s	39, 40, 41
43	197.3, C	10.08, s	32, 33, 34

44	21.1, CH_3	1.10, d (7.5)	25, 26, 27
34-OH		11.73, s	32, 33, 34
37-OH		9.18, s	35, 37

Figure S1. ^{1}H NMR spectrum (700 MHz, DMSO-d_6) of cryptoechinulin B (1)

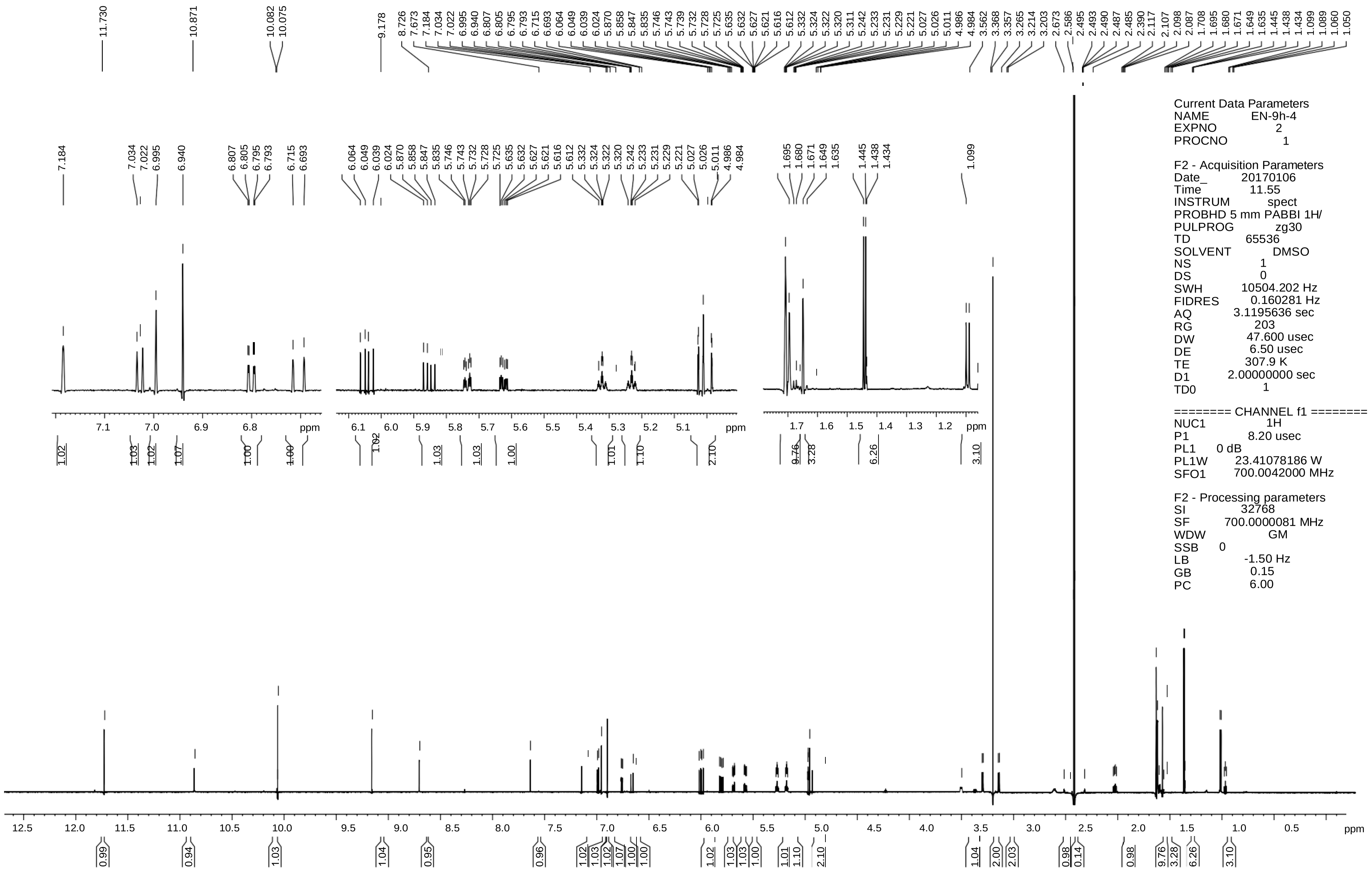

Figure S2. ^{13}C NMR spectrum (176 MHz, DMSO-d_6) of cryptoechinulin B (1)

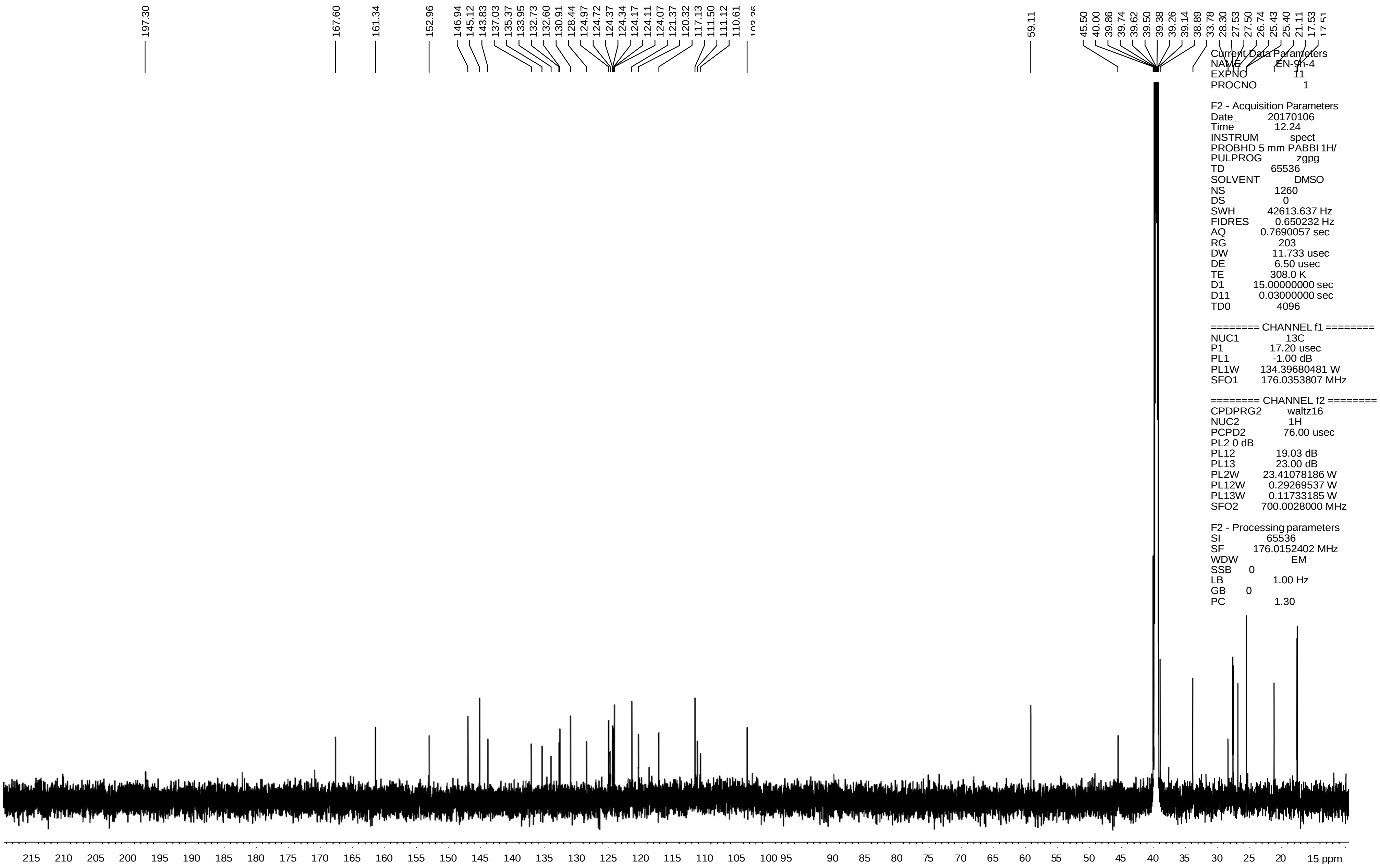

Figure S3. DEPT-135 spectrum (176 MHz, DMSO-d_6) of cryptoechinulin B (1)

Current Data Parameters
NAME EN-9h-4
EXPNO 75
PROCNO 1

F2 - Acquisition Parameters
Date_ 20170202
Time 12.42
INSTRUM spect
PROBHD 5 mm PATXO 31P
PULPROG dept135
TD 65536
SOLVENT DMSO
NS 180
DS 32
SWH 42613.637 Hz
FIDRES 0.650232 Hz
AQ 0.7690057 sec
RG 203
DW 11.733 usec
DE 6.50 usec
TE 308.7 K
CNST2 145.0000000
D1 2.00000000 sec
D2 0.00344828 sec
D12 0.00002000 sec
TD0 4096

======== CHANNEL f1 ========
NUC1 13C
P1 9.10 usec
P2 18.20 usec
PL1 0 dB
PL1W 106.75517273 W
SFO1 176.0353807 MHz

======== CHANNEL f2 ========
CPDPRG2 waltz16
NUC2 1H
P3 16.00 usec
P4 32.00 usec
PCPD2 71.90 usec
PL2 0 dB
PL12 13.05 dB
PL2W 23.41078186 W
PL12W 1.15988755 W
SFO2 700.0028000 MHz

F2 - Processing parameters
SI 65536
SF 176.0152409 MHz
[illegible]
GB 0
PC 1.30

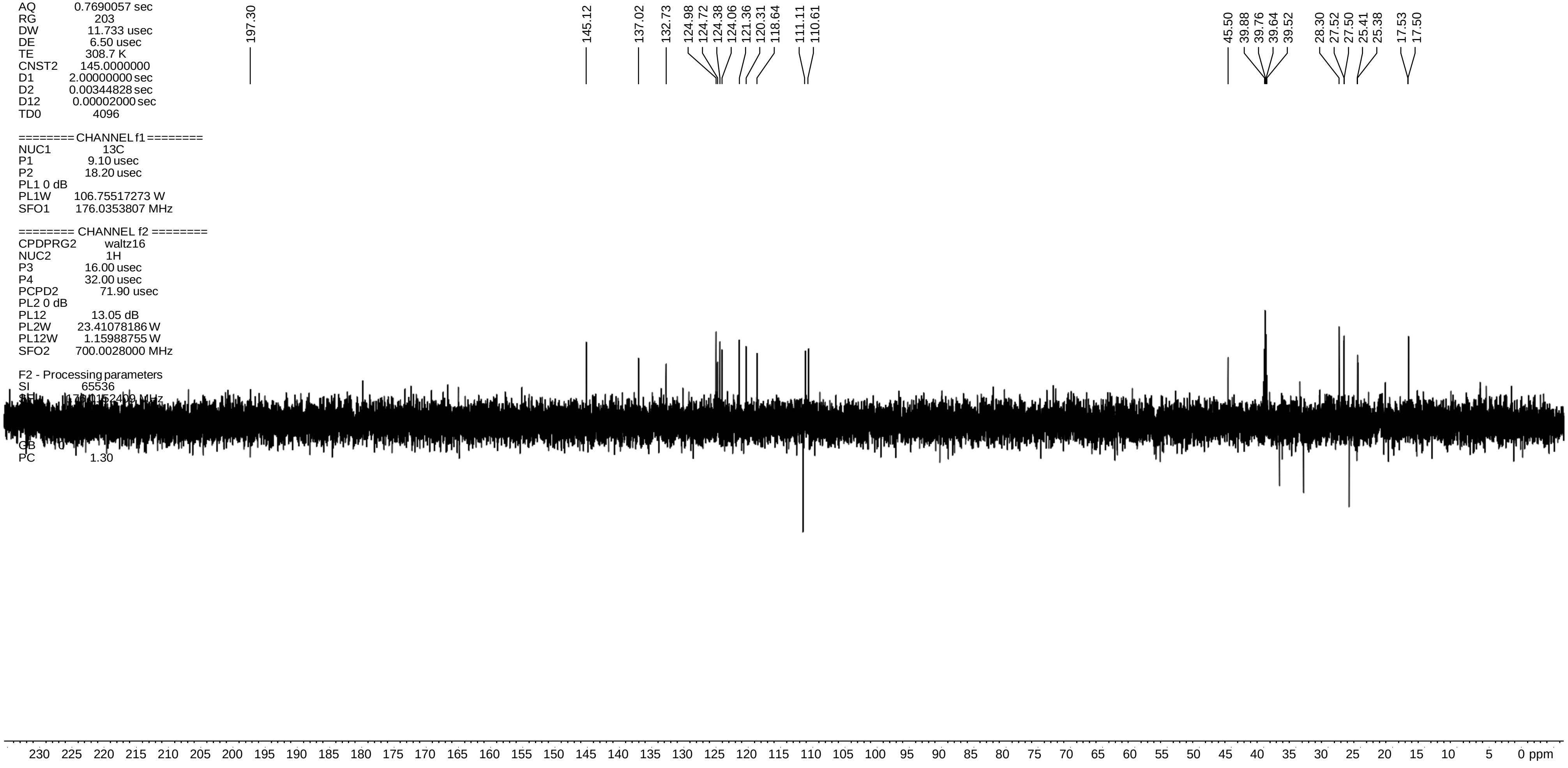

Figure S4. HSQC spectrum (700 MHz, DMSO-d_6) of cryptoechinulin B (1)

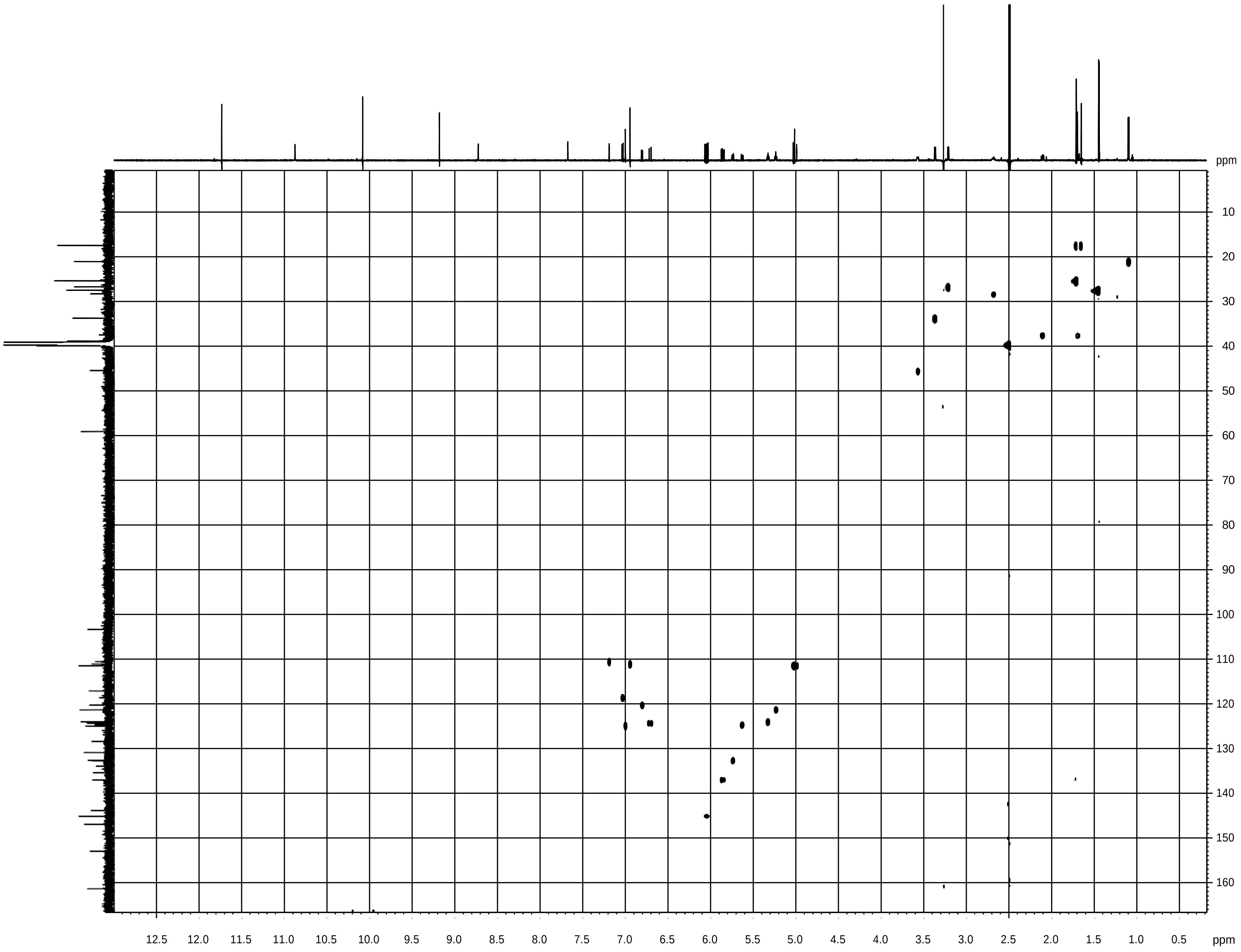

Current Data Parameters
NAME EN-9h-4
EXPNO 22
PROCNO 1

F2 - Acquisition Parameters
Date_ 20170202
Time 15.38
INSTRUM spect
PROBHD 5 mm PATXO 31P
PULPROG hsqcph
TD 1024
SOLVENT DMSO
NS 16
DS 16
SWH 8971.292 Hz
FIDRES 8.761027 Hz
AQ 0.0571209 sec
RG 203
DW 55.733 usec
DE 6.50 usec
TE 308.1 K
CNST2 145.0000000
D0 0.00000300 sec
D1 2.00000000 sec
D4 0.00172414 sec
D11 0.03000000 sec
IN0 0.00001710 sec
L31 1

======== CHANNEL f1 ========
NUC1 1H
P1 14.40 usec
P2 28.80 usec
PL1 0 dB
PL1W 23.41078186 W
SFO1 700.0046159 MHz

======== CHANNEL f2 ========
CPDPRG2 bi_p5m4sp_4sp.2
NUC2 13C
P3 9.20 usec
P4 18.40 usec
P63 1500.00 usec
PL2 0 dB
PL12 16.00 dB
PL2W 106.75517273W
PL12W 2.68156862 W
SFO2 176.0299251 MHz
SP14 10.36 dB
SP31 16.38 dB
SPNAM14 Crp32,1.5,20.2
SPNAM31 Crp32,1.5,20.2
SPOAL14 0.500
SPOAL31 0.500
SPOFFS14 0 Hz
SPOFFS31 0 Hz

F1 - Acquisition parameters
TD 256
SFO1 176.0299 MHz
FIDRES 114.144402 Hz
SW 166.000 ppm
FnMODE TPPI

F2 - Processing parameters
SI 2048
SF 700.0000047 MHz
WDW QSINE
SSB 2
LB 0 Hz
GB 0
PC 4.00

F1 - Processing parameters
SI 512
MC2 TPPI
SF 176.0151979 MHz
WDW
SSB 2
LB 0 Hz
GB 0

Figure S5. HMBC spectrum (700 MHz, DMSO-d_6) of cryptoechinulin B (1)

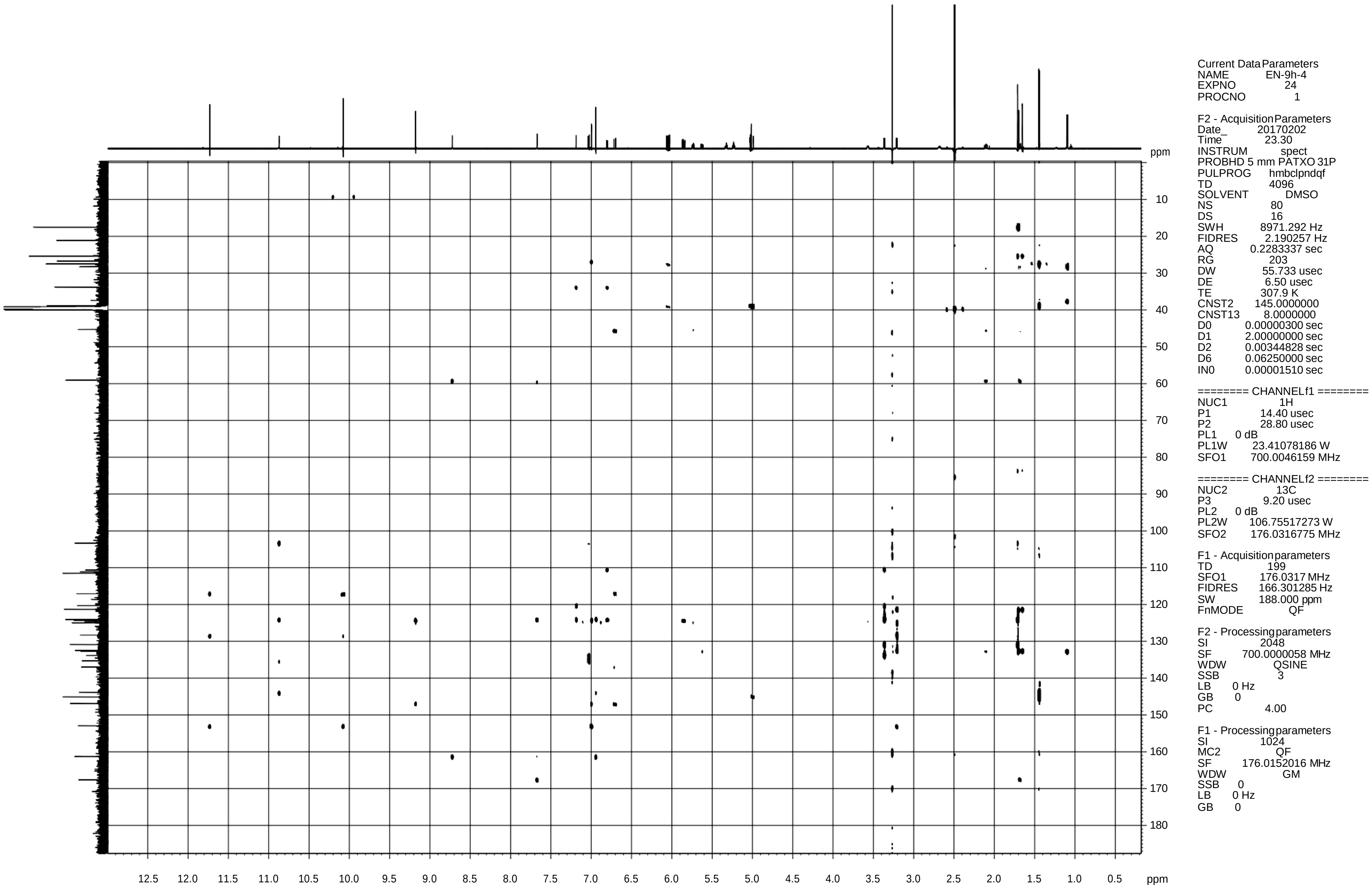

Figure S6. ^{1}H-^{1}H COSY spectrum (700 MHz, DMSO-d_6) of cryptoechinulin B (1)

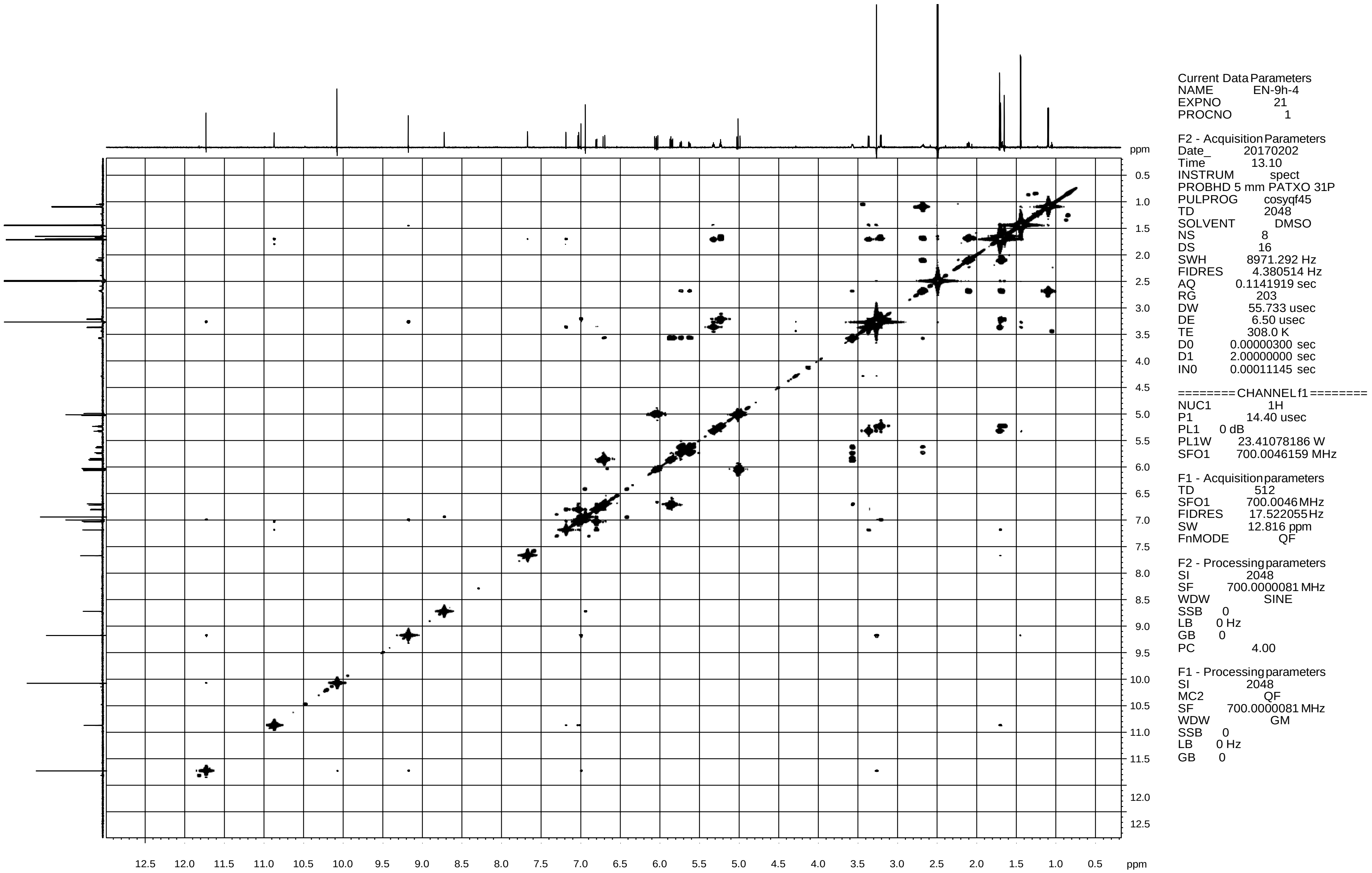

Figure S7. ROESY spectrum (700 MHz, DMSO-d6) of cryptoechinulin B (1)

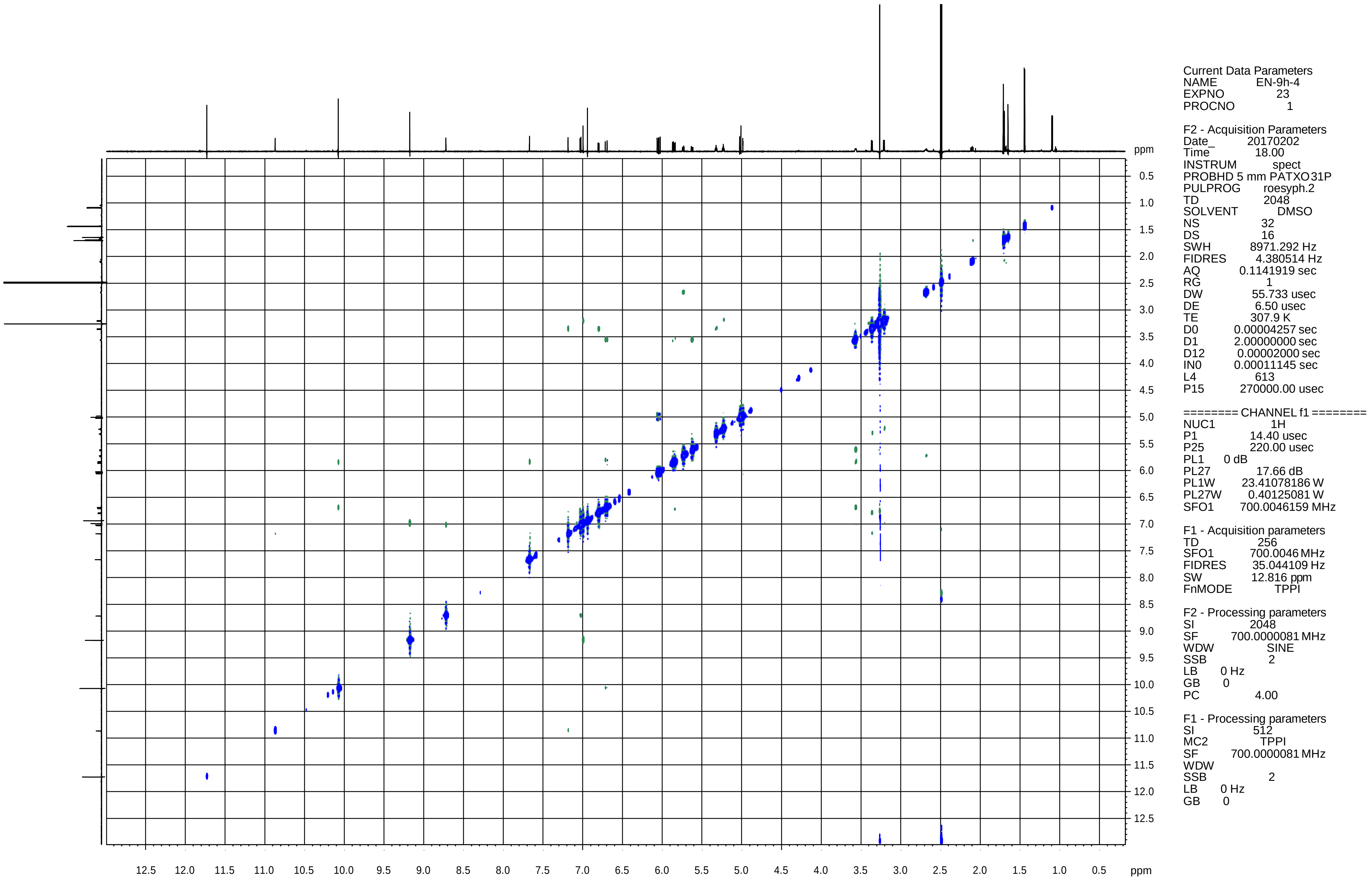

Figure S8. CD spectrum of (+)-cryptoechinulin B (1a) and (–)-cryptoechinulin B (1b)

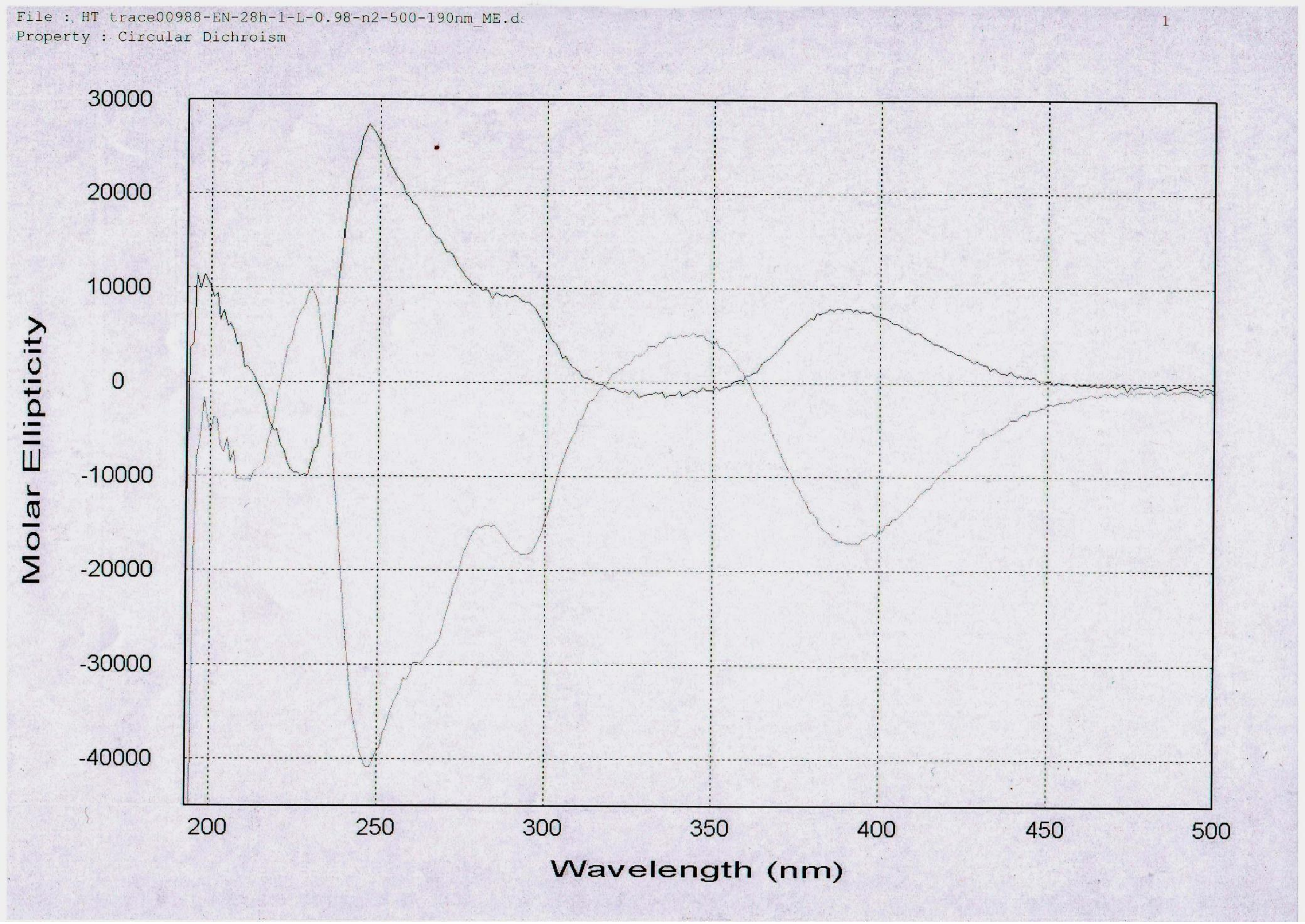

Supplement: Supplementary file 1 [file molecules-25-00061-s001.pdf]
